# STAble: a novel approach to de novo assembly of RNA-seq data and its application in a metabolic model network based metatranscriptomic workflow

**DOI:** 10.1186/s12859-018-2174-6

**Published:** 2018-07-09

**Authors:** Igor Saggese, Elisa Bona, Max Conway, Francesco Favero, Marco Ladetto, Pietro Liò, Giovanni Manzini, Flavio Mignone

**Affiliations:** 1 Dipartimento di Scienze e Innovazione Tecnologica, Università degli Studi del Piemonte Orientale, 15121 Alessandria, Italy; 2 Computer Laboratory, University of Cambridge, Cambridge, CB2 1TN UK; 3 Dipartimento di Scienze della Salute, Università degli Studi del Piemonte Orientale, 28100 Novara, Italy; 4 AO SS Antonio e Biagio e Cesare Arrigo, 15121 Alessandria, Italy; 5 Istituto di Informatica e Telematica, CNR, 56124 Pisa, Italy; 6 Dipartimento di Biotecnologie e Scienze per la Salute, Università di Torino, 10124 Torino, Italy

## Abstract

**Background:**

De novo assembly of RNA-seq data allows the study of transcriptome in absence of a reference genome either if data is obtained from a single organism or from a mixed sample as in metatranscriptomics studies. Given the high number of sequences obtained from NGS approaches, a critical step in any analysis workflow is the assembly of reads to reconstruct transcripts thus reducing the complexity of the analysis. Despite many available tools show a good sensitivity, there is a high percentage of false positives due to the high number of assemblies considered and it is likely that the high frequency of false positive is underestimated by currently used benchmarks. The reconstruction of not existing transcripts may false the biological interpretation of results as – for example – may overestimate the identification of “novel” transcripts. Moreover, benchmarks performed are usually based on RNA-seq data from annotated genomes and assembled transcripts are compared to annotations and genomes to identify putative good and wrong reconstructions, but these tests alone may lead to accept a particular type of false positive as true, as better described below.

**Results:**

Here we present a novel methodology of de novo assembly, implemented in a software named STAble (Short-reads Transcriptome Assembler). The novel concept of this assembler is that the whole reads are used to determine possible alignments instead of using smaller k-mers, with the aim of reducing the number of chimeras produced. Furthermore, we applied a new set of benchmarks based on simulated data to better define the performance of assembly method and carefully identifying true reconstructions.

STAble was also used to build a prototype workflow to analyse metatranscriptomics data in connection to a steady state metabolic modelling algorithm. This algorithm was used to produce high quality metabolic interpretations of small gene expression sets obtained from already published RNA-seq data that we assembled with STAble.

**Conclusions:**

The presented results, albeit preliminary, clearly suggest that with this approach is possible to identify informative reactions not directly revealed by raw transcriptomic data.

## Background

Among many applications of Next Generation Sequencing (NGS), [1] there are two techniques that can be applied to the “omic” study of transcripts: RNA-seq [2] that profiles transcriptomes from a single organism or metatranscriptomics that profiles transcriptomes from a complex microbial community.

The first field is more established and allows to assess the presence of RNA transcripts in a biological sample at a given moment and to perform quantification. The latter is a more recent and less explored approach related to metagenomics studies: while metagenomics aims at the identification of species, metatranscriptomics tries to characterize functional active bacteria and their metabolic interaction through the identification of the expressed transcripts.

Facing the growing promises and challenges of clinical metagenomics, metatranscriptomics analysis might represent a critical step to further elucidate the role of complex microbial communities in the physiology and pathology of host organisms with a growing impact in clinical application. Indeed, most of the evidence so far accumulated is linked to the role of specific species, genera or families rather that to their metabolic output. While this might be optimal in terms of impact on immune recognition, immune education and trigger of autoimmune processes, this approach may be insufficient to fully elucidate the impact of microbial communities on processes such as metabolic diseases, inflammatory response, and nutrient availability which are potentially more strictly related to the global metabolic output rather than to the phylogenesis of the species composing a specific microbiota.

From the perspective of data analysis, current NGS sequencing platforms do not output the whole transcripts but short reads representing a fragment of the original sequence. Assembly of reads to reconstruct full transcripts represents a crucial point in data analysis and any subsequent steps in the analysis of transcriptomics data heavily rely on the quality of reconstructions. Even when a reference genome is available for the organism under study, the preventive assembly of reads can prove useful to reduce the complexity of the analysis by both increasing the length and lowering the number of input sequences. Currently state-of-the-art tools to reconstruct RNA-seq data are Bridger [3], Oases [4] and Trinity [5]. They share a similar approach as they rely on the identification of k-mer sequences. Bridger then uses this information to build and traverse splicing graphs, while Oases and Trinity rely on De-Bruijn graphs.

Despite exhibiting a good sensitivity, all of them show two main limitations: i) high number of false positive reconstructions and ii) very high demands of computational power.

Working with real data, in absence of any reference, it is not trivial - and maybe not even possible - to determine the correctness of a reconstruction, so it is advisable to use approaches that minimize the production of false reconstructions. High sensitivity claimed in benchmarks is often obtained by increasing the number of reconstructions, at the cost of increasing the number of false positives too, but this aspect is usually neglected. Furthermore, current approaches are very demanding in terms of hardware specifications and dedicated infrastructures are required but they are not always available.

**Fig. 1:**
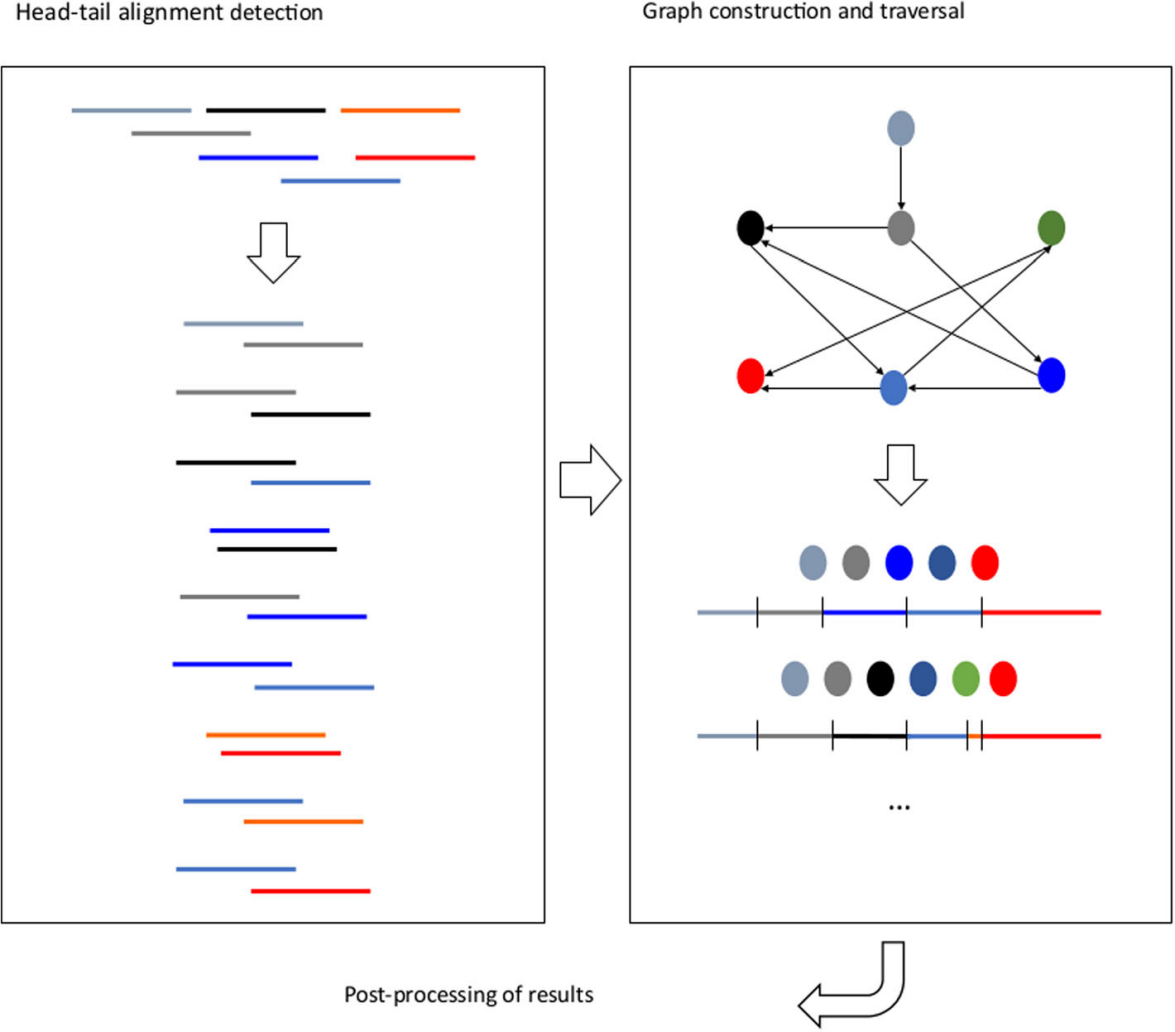
STAble’s analysis workflow. The first module detects potential head-tail alignments between reads, the second one uses this information to build and traverse a directed unweighted graph to reconstruct transcripts that are then post-processed before returning final output

Here we present STAble, a prototype for a new de novo assembler developed around a novel approach quite different from the state-of-the-art: the whole reads are used to determine possible alignments instead of using smaller k-mers, with the aim of drastically reduce the number of chimeras produced. STAble consists of three different modules (see Fig. 1). The first step is the efficient detection of potential head-tail alignments between reads, possibly with mismatches. This information is then used by the second module to build an unweighted directed graph, which is traversed by a custom algorithm that takes into account biological properties of input data. Finally, the third module performs some post-processing on results assuming no reference information is available.

In benchmarks, STAble has shown a sensitivity comparable to current tools, while producing a smaller number of false positive reconstructions. STAble is designed to be parallelizable and grid-friendly, allowing to split input datasets in blocks that can be processed sequentially or in parallel computations: this feature allows to perform analyses even in absence of dedicated computing infrastructures. Moreover, STAble was tested with both simulated and real metatranscriptomics data. With simulated data we were able to evaluate the ability of our system to correctly reconstruct transcripts while with real data we tested a prototype implementation of a new approach based on the integration of transcriptomics data with metabolic network.

## Methods

STAble implements an original approach based on the idea to let the whole reads guide the assembly process, instead of considering smaller k-mers with the aim of reducing false positive reconstructions. Analysis workflow is shown in Fig. 1 and consists of three main modules:

1. Efficient detection of head-tail alignments.
2. Construction and traversal of an unweighted directed graph.
3. Post processing of results.

The first module identifies overlapping reads: it starts from a fastq file containing input sequences and finds all “valid” head-tail overlaps between pairs of reads. More precisely, the module is based on a custom procedure to identify head-tail overlaps that works as follow. Computation starts by recoding input FASTQ from 8-bit ASCII characters to a 2-bit alphabet: this allows a reduction in memory consumption and speeds up subsequent operations. No special symbol is assigned to ambiguous bases - such as N - but the same symbol reserved for C is used. This choice was made to keep the size of the new alphabet as low as possible. Results quality is not affected since reads with too many ambiguous bases are usually discarded by pre-processing steps because of low quality, so false matches with C are expected to be rare.

After initialisation is done, the algorithms proceed to analyse input sequences one at a time and each 7 nt long anchor is indexed. The first and last *anchor scope* (default: 5) anchors are searched in the anchor index to detect potential aligning reads. Read pairs are then shifted to align the anchor and Hamming distance of the overlapping area is efficiently computed as number of mismatches by using XOR metrics. The module returns a list of triples [*i; j; k*] where *i* and *j* represent two reads and *k* is the length of the overlap found between the tail of read *i* and the head of read *j*. A head-tail overlap is considered “valid” only if it satisfies the following two conditions:

1. Hamming distance between the length-k tail of sequence i and the length-k head of sequence j must not be greater than max_errors, where max_errors is the maximum number of mismatches allowed. (default: 10% of overlap length).
2. Overlap length k is a value between min_len and max_len. min_len is the minimum length allowed for overlaps (default: 20% of longer sequence between overlapping pair) and max_len is the maximum length allowed for overlaps (default: 90% of shorter sequence between overlapping pair). Although RNA-seq reads are supposed to have all the same length, our algorithm can work even on reads with different lengths. This is useful if sequences have been previously quality filtered.

The first condition is pivotal to guarantee a good alignment and avoid the reconstruction of chimeric transcripts.

Regarding the second condition, a minimum length for the overlap is required to avoid alignments caused by casual similarities.

Similarly, a maximum length must be set to deal with redundancy of information caused by high sequencing depths: an alignment caused by an excessive overlap will generate a poorly informative contig (just “few” bases longer than the single read).

The triples returned by the first module are used to build an unweighted directed graph *G* where each node represents a read and an arc a head-tail alignment between two reads. Ideally, every path in *G* from a source (node without incoming edges) to a sink (node without outgoing edges) would represent a transcript, or a fragment of it. However, due to the high sequencing depths the same transcript or fragment could be obtained by many paths differing for a small number of nodes and it would be too expensive to generate all of them. In addition, the presence of alternative splicing and head-tail alignments over repeated regions may lead to chimeric reconstructions. To take into account all these issues we have developed a custom traversal algorithm, which is the core of the second module.

The traversal algorithm executes a depth-first search starting from each source node in *G. W*hen a sink node is reached, the current path is output if its length is greater than the parameter *minLenght*. During the depth-first search we discard the current path if it turns out to be “too similar” to a prefix of an already generated path originating from the same source. For this purpose, two paths are considered “too similar” if they have the same first and last nodes, and one path can be obtained from the other replacing at most *simThreshold* nodes. Another technique to reduce the number of paths produced by the traversal algorithm is to enforce that each path should contain a minimum number of “new” nodes. This is achieved as follows. Initially all nodes are colored white. When a path is output all its nodes are colored black, and we output a new path only if it contains at least *whiteThreshold* nodes. At the end of the graph traversal, all produced paths are transformed into transcripts by replacing each node with the read it represents and combining the reads keeping into account the length of their overlaps. This set of transcripts is the output of the second module.

Finally, the third module processes the resulting transcripts are processed by performing various operations: the most important one is the clustering of sequences to remove the last degree of redundancy that is not detected by traversal algorithm.

The last module performs a post-processing removal of redundancies by using clustering algorithms. Currently we implement Usearch algorithm [6] for a fast removal of duplicated sequences.

Finally, all reconstructed transcripts are weighted by a quick bowtie alignment with raw reads.

STAble is designed to be parallelizable and grid-friendly in order to speed up analysis process and reduce hardware requirements. The idea is to random split input dataset in smaller blocks of size k: each block is then processed with the three modules described above. Processing of each block can be performed sequentially or in parallel computations even on common desktop computers. Results are then merged, clustered and used as input for a new iteration: computation stops when dataset size becomes smaller than k.

### Known limitations

Current version of STAble suffers from some known limitations. First it treats paired-end reads as single-end and does not takes advantages of the information provided by the paired end approach. Moreover, the head-tail alignment of reads does not manage reverse complement pairing. This leads to the redundant identification of each transcript in both forward and reverse strand. This issue is minimised by the post-processing clustering applied but it would be advisable to upgrade the analysis procedure to correctly handle reverse complement pairing with an expected improvement of reconstructions.

### Benchmark

Simulated datasets were generated selecting random transcripts from human genome or from bacteria and producing reads using ART [7] as Illumina 150 bp single end with 20× of fold coverage and HiSeq 2500 quality profile. Reads were used to reconstruct transcripts with STAble and with other assemblers (default parameters were used). Reconstructed transcripts were aligned to database used for simulations using BLASTn: reconstructed transcripts not aligning as a single match for at least 85% of its length to any reference sequence were marked as False Positives. False Positives were then aligned to genome with GMAP [8]. If the mapping showed a realistic pattern of introns-exons the reconstructed transcript was labelled as False Positive class A – FPA, A match was considered “realistic” if resulting from GMAP analysis as a single path covering 90% of the transcript with at least 90% of similarity. False positive reconstructions not satisfying these criteria were labelled as False Positive class B - FPB (see results for details).

True Positives transcripts reconstructing reference sequences for at least 90% of their length were labelled as full-length reconstructed.

### Hardware

STAble was run on a desktop computer equipped with a dual-core Intel Core i3 processor and 8GB of RAM. Other tools were tested on an Intel Xeon with 8 cores and 48GB of RAM.

### Real datasets

**Fig. 2:**
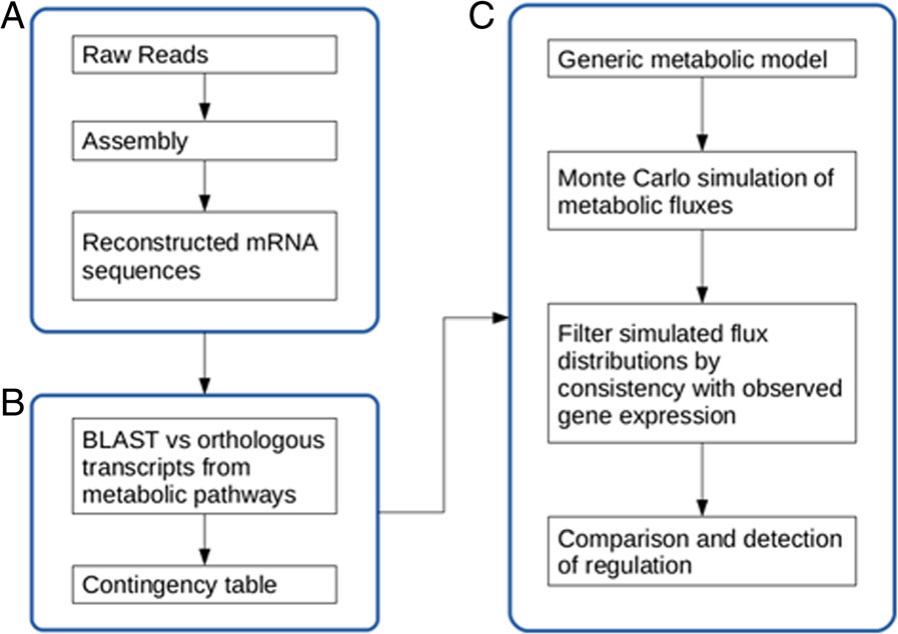
Workflow of metatranscriptomic analysis integrated with metabolic network. **a** Raw reads from were assembled with a default STAble analysis. **b** Reconstructed transcripts were assigned to orthologous transcripts included in several metabolic pathways as annotated in KEGG database. A contingency table with KEGG reference genes binned with reconstructed transcripts is generated. **c** Metabolic model flux analysis to interpret gene expression using the method described in [13]

Raw data described in [9] were downloaded from National Centre for Biotechnology Information Sequence Read Archive, accession number SRA075938, bioproject number PRJNA202380 [10]. We downloaded a total number of six metatranscriptomic samples with the following names according to [9] Sheep tag: S1234 = SRR1206249 (high), S1494 = SRR873453 (low), S1333 = SRR873463 (high), SRR1283 = SRR873451 (low), S1265 = SRR873454 (low), S1586 = SRR873461 (high). Raw datasets were downloaded in fastq format and used as input for our analysis workflow. The first step was the assembly of reads with STAble to reconstruct transcripts. We then downloaded bacterial FASTA sequences of orthologous genes of several pathways (glycolysis/gluconeogenesis, butanoate metabolism, methane metabolism, carbon fixation pathways, phosphotransferase system) from KEGG ortholog database [11]. Reconstructed transcripts were aligned to bacterial genes using BLAST accepting matches with at least 92% of similarity and allowing up to 20 nucleotides of mismatches over flanking regions. The contingency tables with read count for each orthologous gene were processed with metabolic models to interpret gene expression. The method adopted is described in [12]. Briefly, we performed a blind Monte-Carlo simulation over feasible flux configurations. Specifically, we sampled from the set of flux configurations that provide near optimal biomass, while also providing optimality against a second random set of objectives. We then regard this large set of flux configurations as the set of possible populations (G), and then find the subset (termed L) of G which is consistent with the experimentally determined gene expression vectors. This is achieved by gene-by-gene parametric comparison between G and the set of gene expression vectors. Finally, we compare L to G to understand which reactions are most strongly influenced by the gene expressions tested. The overall method is depicted in Fig. 2.

## Results and discussion

STAble performance was compared with Bridger, Oases and Trinity. The prototype was tested on a large set of simulated data in order to be able to perform deeper evaluations on results quality. Benchmarks are usually performed on real data, using RNA-seq data from organisms for which a reference genome is available. Reconstructed transcripts are then compared with annotated transcripts to identify good quality reconstructions. By aligning reconstructed transcripts with genome it is possible to identify chimeric or unrealistic transcripts (i.e. mapping onto multiple chromosomes, with unlikely long introns or with inversions).

**Fig. 3:**
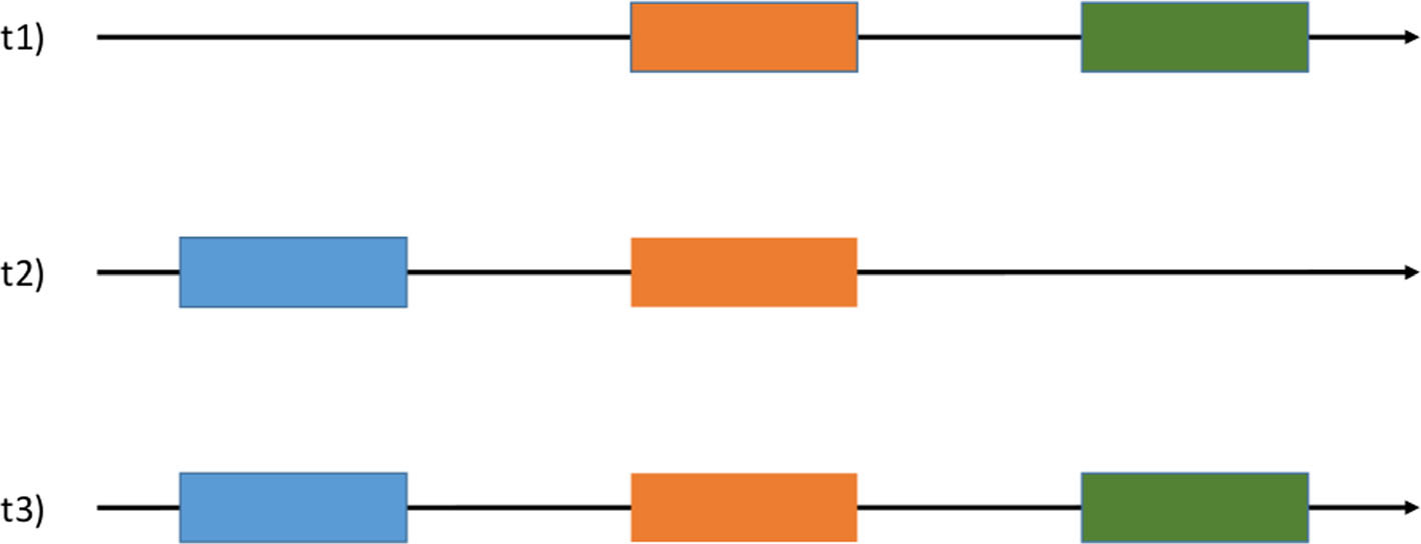
Supposing that only splicing variants t1 and t2 are present in the sample, reads may allow to reconstruct t3 that is a valid annotated alternative but has to be considered as a false positive

We benchmarked STAble with simulated data because they allow the unambiguous identification of true and false assembled transcripts which is only partially possible with real datasets. By working with simulated datasets we highlighted a new kind of false positive reconstruction which is not visible with real data. This false positive type (we named False Positive class A - FPA) is depicted in Fig. 3. Let’s suppose that t1, t2 and t3 are annotated alternative splicing forms of the same gene and that only t1 and t2 are present in sample: reads may allow to reconstruct t3 even if it is not effectively transcribed, so t3 has to be considered as a false positive. However, with real data it is not be possible to identify FPA (as t3 is a real transcript albeit not expressed in the sample under analysis) so the rate of false positives is likely to be underestimated.

In the following discussion we label as FPA (False Positive class A) false positive reconstructions that do not match any sequence in the database used for simulation but correctly match with genome, while we label as FPB (False Positive class B) chimeric reconstructions.

Simulated data and analysis were performed as described in Methods.

Table 1 summarizes the results obtained assembling 147,800 simulated reads from a pool of 200 transcripts and 1,088,271 reads from a pool of 6309 transcripts randomly picked from human transcriptome. Results show that STAble performs similarly with other tools in term of sensitivity. While Oases and Trinity show a slightly higher number of transcripts reconstructed at 100% it has to be noted that they are affected by a high rate of false positives. Bridger and Oases show the highest rate of FPB, Oases and Trinity show a very high number of FPA. Only STAble performs reconstructions with a low rate of both FPA and FPB. Moreover, it is important to underline that when considering reference transcripts reconstructed at least 70% STAble performance is almost the same as Trinity’s. It is interesting to note that on benchmark datasets based on real data with reference genome (for which a real set of actually expressed sequences is not available) - where it is not possible to detect FPA Trinity would have shown a very low false positive rate as FPA would have been detected as True Positives.

**Table 1:** Results on 200 (Dataset A) and 6309 (Dataset B) random human transcripts. STAble returned the most reliable set of results showing a sensitivity comparable to other assemblers while producing only 3 false positives

Dataset A									
Assembler	# of results	# of FP	FPA	FPB	100%	70%	S100	S70	FPR
STAble	227	1	0	1	152	161	76%	81%	0.44%
Bridger	210	58	30	28	143	148	72%	74%	28%
Oases	321	106	89	17	159	165	80%	83%	33%
Trinity	258	56	48	8	157	167	79%	84%	22%
Dataset B									
Assembler	# of results	# of FP	FPA	FPB	100%	70%	S100	S70	FPR
STAble	8906	2285	1053	1232	3295	4179	52%	66%	26%
Bridger	5697	1820	945	875	2728	3315	43%	53%	32%
Oases	16,895	5722	2835	2887	3550	4156	56%	66%	34%
Trinity	8300	2543	2223	320	3603	4315	57%	68%	31%

*Assembler* Name of the assembler, *# of results* Total number of reconstructed transcripts, *# of FP* Number of False Positive results, *FPA* False Positive class A, *FPB* False Positive class B, *100%* Number of full reconstructed transcripts, *70%* Number of transcripts reconstructed at 70%, *S100* Percentage of full reconstructed transcripts, *S70* Percentage of transcripts reconstructed at 70%, *FPR* False Positive Ratio

Finally, we performed some benchmarks on simulated bacterial metatranscriptomic datasets. Annotated transcripts from 10 different species were mixed and used to generate two additional simulated datasets: 1242040 reads from a pool of 11,815 mixed bacterial transcripts, and 2,382,790 reads from a pool of 43,578 mixed bacterial transcripts. Results are summarized in Table 2. STAble has shown the highest sensibility with a comparable FPR with the other programs. It is interesting to note that due to absence of alternative splicing in bacterial transcriptome it is not possible to produce FPA class errors (see Table 2). Noticeably STAble - running on a desktop computer equipped with 8GB of RAM - was the only assembler capable of completing the assembly task with the larger dataset. All existing tools terminated returning an out of memory error even on a computer with 48GB of RAM.

**Table 2:** Eleven thousand eight hundred fifteen (dataset C) and 43,578 (dataset D) mixed bacterial transcripts. STAble shown the best sensitivity while producing the lowest false positive ratio alongside with Trinity. Due to absence of alternative splicing in bacterial transcriptome it is not possible to produce FPA class errors. With the larger dataset it is not possible to compare results with existing assemblers as they terminated with an out of memory error

Dataset C							
Assembler	# of results	# of FP	100%	70%	S100	S70	FPR
STAble	13,985	983	10,007	10,263	85%	87%	7%
Bridger	5873	253	8510	9075	72%	77%	4%
Oases	5579	268	6687	8603	57%	73%	5%
Trinity	7597	145	9136	9565	77%	81%	2%
Dataset D							
Assembler	# of results	# of FP	100%	70%	S100	S70	FPR
STAble	134,110	1040	20,800	35,424	48%	81%	0.8%

*Assembler* Name of the assembler, *# of results* Total number of reconstructed transcripts, *# of FP* Number of False Positive results, *FPA* False Positive class A, *FPB* False Positive class B, *100%* Number of full reconstructed transcripts, *70%* Number of transcripts reconstructed at 70%, *S100* Percentage of full reconstructed transcripts, *S70* Percentage of transcripts reconstructed at 70%, *FPR* False Positive Ratio

To test our workflow on real data we took advantage of the work by Kamke and colleagues [9]. In their paper, they make a comparison of rumen microbiome of high and low methane yield sheep with metatranscriptomic studies. We downloaded raw reads from SRA for 3 high and 3 low methane yield samples and we processed them as described in Materials and Methods, then we compared our results with the ones discussed by Kamke and colleagues [9]. Briefly, reads were assembled with STAble, mapped to KEGG orthologous genes of few basics bacteria metabolic pathways. The usage of few metabolic pathways instead of the more time-consuming usage of the entire genes set is consistent with our approach. Indeed, as described in Materials and Methods the metabolic flux algorithm used can work well even with small gene expression sets. In particular, we compared reconstructed transcripts with genes involved in some metabolic pathway such as the glycolysis/gluconeogenesis pathway (as an example of basic bacterial metabolism pathway), the butanoate metabolism and the methane metabolism pathway. We also used the carbon fixation pathways in prokaryotes and the membrane transport pathway of phosphotransferase system that is one of the pathway cited and analysed by Kamke and colleagues [9].

The contingency tables with genes and their abundance were used to feed a metabolic model network to interpret gene expression. The simplest approach when performing this kind of analysis is to directly design a mapping function, which projects the gene expressions as constraints on their associated reactions in the metabolic model. This gives a one to one mapping between gene expression vectors and metabolic models, and necessitates a great degree of care in the design of the mapping function. Specifically, the mapping function needs to produce detectable differences between metabolic models, while also ensuring that predicted fluxes are all within the bounds of what is biologically feasible.

Here, we take a radically different approach. Rather than parameterizing metabolic models using gene expression vectors directly, we instead perform a blind Monte-Carlo simulation over flux configurations that provide near optimal biomass. We then regard this large set of flux configurations as the set of possible populations (G), and then find the subset (termed L) of G, which is consistent with the experimentally determined gene expression vectors. Finally, we compare L to G to understand which reactions are most strongly influenced by the gene expressions tested (summarized in Table 3 and Table 4).

**Table 3:** List of all bacterial metabolic reactions identified in high methane yield animals

Abbreviation	Subsystem	Official Name
NADH16pp	Oxidative Phosphorylation	NADH dehydrogenase (ubiquinone-8 & 3 protons) (periplasm)
PROt2rpp	Transport	L-proline reversible transport via proton symport (periplasm)
PROt4pp	Transport	Na+/Proline-L symporter (periplasm)
GLCP2	Glycolysis/Gluconeogenesis	glycogen phosphorylase
GLCS1	Glycolysis/Gluconeogenesis	glycogen synthase (ADPGlc)
GLGC	Glycolysis/Gluconeogenesis	glucose-1-phosphate adenylyltransferase
THRt2rpp	Transport	L-threonine reversible transport via proton symport (periplasm)
THRt4pp	Transport	L-threonine via sodium symport (periplasm)
INSt2pp	Transport	inosine transport in via proton symport (periplasm)
INSt2rpp	Transport	inosine transport in via proton symport reversible (periplasm)
PPCSCT	Alternate Carbon Metabolism	Propanoyl-CoA: succinate CoA-transferase
SUCOAS	Citric Acid Cycle	succinyl-CoA synthetase (ADP-forming)
TALA	Pentose Phosphate Pathway	transaldolase
ACCOAL	Alternate Carbon Metabolism	acetate-CoA ligase (ADP-forming)
GLUt4pp	Transport	Na+/glutamate symport (periplasm)
PPAKr	Alternate Carbon Metabolism	Propionate kinase
PTA2	Alternate Carbon Metabolism	Phosphate acetyltransferase
THFAT	Folate Metabolism	Tetrahydrofolate aminomethyltransferase
FOMETRi	Folate Metabolism	Aminomethyltransferase
ADK3	Nucleotide Salvage Pathway	adentylate kinase (GTP)
FBA3	Pentose Phosphate Pathway	7-bisphosphate D-glyceraldehyde-3-phosphate-lyase
PFK_3	Pentose Phosphate Pathway	phosphofructokinase (s7p)
URAt2pp	Transport	uracil transport in via proton symport (periplasm)
URAt2rpp	Transport	uracil transport in via proton symport reversible (periplasm)
GLYt2pp	Transport	glycine transport in via proton symport (periplasm)
GLCP	Glycolysis/Gluconeogenesis	glycogen phosphorylase
NDPK1	Nucleotide Salvage Pathway	nucleoside-diphosphate kinase (ATP:GDP)
CA2t3pp	Inorganic Ion Transport and Metabolism	calcium (Ca + 2) transport out via proton antiport (periplasm)
CAt6pp	Inorganic Ion Transport and Metabolism	calcium / sodium antiporter (1:1)
PPKr	Oxidative Phosphorylation	polyphosphate kinase
URIt2pp	Transport	uridine transport in via proton symport (periplasm)
URIt2rpp	Transport	uridine transport in via proton symport reversible (periplasm)
NADH18pp	Oxidative Phosphorylation	NADH dehydrogenase (demethylmenaquinone-8 & 3 protons) (periplasm)
FRD3	Citric Acid Cycle	fumarate reductase
ALAt2pp	Transport	L-alanine transport in via proton symport (periplasm)
ALAt2rpp	Transport	L-alanine reversible transport via proton symport (periplasm)
GLYt2rpp	Transport	glycine reversible transport via proton symport (periplasm)

**Table 4:** List of all bacterial metabolic reactions identified in low methane yield animals

Abbreviation	Subsystem	Official Name
ALATA_L	Alanine and Aspartate Metabolism	L-alanine transaminase
THMDt2pp	Transport	thymidine transport in via proton symport (periplasm)
THMDt2rpp	Transport	thymidine transport in via proton symport reversible (periplasm)
NAt3pp	Inorganic Ion Transport and Metabolism	sodium transport out via proton antiport (cytoplasm to periplasm)
VPAMTr	Valine, Leucine and Isoleucine Metabolism	Valine-pyruvate aminotransferase
VALTA	Valine, Leucine and Isoleucine Metabolism	valine transaminase
SUCDi	Oxidative Phosphorylation	succinate dehydrogenase (irreversible)
GLUABUTt7pp	Transport	4-aminobutyrate/glutamate antiport (periplasm)
ABUTt2pp	Transport	4-aminobutyrate transport in via proton symport (periplasm)
GLYt4pp	Transport	glycine transport in via sodium symport (periplasm)
GLUt2rpp	Transport	L-glutamate transport via proton symport reversible (periplasm)
GLDBRAN2	Glycolysis/Gluconeogenesis	glycogen debranching enzyme (bglycogen - > glycogen)
GLYCLTt2rpp	Transport	glycolate transport via proton symport
GLYCLTt4pp	Transport	glycolate transport via sodium symport (periplasm)
ACt2rpp	Transport	acetate reversible transport via proton symport (periplasm)
ACt4pp	Transport	Na+/Acetate symport (periplasm)
ADK1	Nucleotide Salvage Pathway	adenylate kinase
PTAr	Pyruvate Metabolism	phosphotransacetylase
ACKr	Pyruvate Metabolism	acetate kinase
ACS	Pyruvate Metabolism	acetyl-CoA synthetase
SERt2rpp	Transport	L-serine reversible transport via proton symport (periplasm)
SERt4pp	Transport	L-serine via sodium symport (periplasm)
GLCtex	Transport	glucose transport via diffusion (extracellular to periplasm)
PRPPS	Histidine Metabolism	phosphoribosylpyrophosphate synthetase
PPM	Alternate Carbon Metabolism	phosphopentomutase
R15BPK	Alternate Carbon Metabolism	Ribose-1,5 bisphosphokinase
R1PK	Alternate Carbon Metabolism	ribose 1-phosphokinase
GLCtexi	Transport	D-glucose transport via diffusion (extracellular to periplasm) irreversible
ADNt2pp	Transport	adenosine transport in via proton symport (periplasm)
ADNt2rpp	Transport	adenosine transport in via proton symport reversible (periplasm)
ASPt2pp	Transport	L-aspartate transport in via proton symport (periplasm)
ASPt2rpp	Transport	L-aspartate transport in via proton symport (periplasm) reversible
INDOLEt2pp	Transport	Indole transport via proton symport irreversible (periplasm)
INDOLEt2rpp	Transport	Indole transport via proton symport reversible (periplasm)
FBA	Glycolysis/Gluconeogenesis	fructose-bisphosphate aldolase
PFK	Glycolysis/Gluconeogenesis	phosphofructokinase
ICHORS	Cofactor and Prosthetic Group Biosynthesis	isochorismate synthase
ICHORSi	Cofactor and Prosthetic Group Biosynthesis	Isochorismate Synthase
HPYRI	Alternate Carbon Metabolism	hydroxypyruvate isomerase
HPYRRx	Alternate Carbon Metabolism	Hydroxypyruvate reductase (NADH)
TRSARr	Alternate Carbon Metabolism	tartronate semialdehyde reductase
CYTDt2pp	Transport	cytidine transport in via proton symport (periplasm)
CYTDt2rpp	Transport	cytidine transport in via proton symport reversible (periplasm)
FRD2	Citric Acid Cycle	fumarate reductase
NADH17pp	Oxidative Phosphorylation	NADH dehydrogenase (menaquinone-8 & 3 protons) (periplasm)
EX_h(e)	Exchange	H+ exchange
EX_fe3(e)	Exchange	Fe3+ exchange
EX_fe2(e)	Exchange	Fe2+ exchange
Htex	Transport	proton transport via diffusion (extracellular to periplasm)
FEROpp	Inorganic Ion Transport and Metabolism	ferroxidase
FE3tex	Transport	iron (III) transport via diffusion (extracellular to periplasm)
FE2tex	Transport	iron (II) transport via diffusion (extracellular to periplasm)
GLBRAN2	Glycolysis/Gluconeogenesis	4-alpha-glucan branching enzyme (glycogen - > bglycogen)
EX_o2(e)	Exchange	O2 exchange
EX_h2o(e)	Exchange	H2O exchange
O2tex	Transport	oxygen transport via diffusion (extracellular to periplasm)
H2Otex	Transport	H2O transport via diffusion (extracellular to periplasm)
CRNDt2rpp	Transport	D-carnitine outward transport (H+ antiport)
CRNt2rpp	Transport	L-carnitine outward transport (H+ antiport)
CRNt8pp	Transport	L-carnitine/D-carnitine antiporter (periplasm)
ALAt4pp	Transport	L-alanine transport in via sodium symport (periplasm)

Results obtained from our metabolic network analysis are consistent with data about differences in usage of Glycolysis/Gluconeogenesis and Butanoate Biosyntesis pathways described in the paper (data not shown). Interestingly our analysis identified new pathways that are independent from the original set of transcripts used to feed the metabolic model network. Indeed, our metabolic network analysis identified that both in LMY and HMY bacteria, transport channels are highly expressed.

STAble can improve data about gene coding for transport membrane proteins and for nutrient (Fe, Ca and Na) transport in bacterial cells both in LMY and HMY, comparing results with those obtained by Kamke and coworkers [9]. Moreover, the performed analysis revealed carbohydrate metabolism as dominating followed by amino acid metabolism, results in agreement with those reported by Hinsu and colleagues that described functionally active bacteria and their biological processes in rumen of buffalo (*Bubalus bubalis*) adapted to different dietary treatments [13].

These results are intriguing because they confirm that our workflow appears to produce more punctual information regarding metabolic pathways upregulated or downregulated into the same microbiome, not directly correlated with the transcripts, identified with raw RNA-seq data.

Our results highlight the potential of our new approach to de novo assembly of RNA-seq data. STAble’s sensitivity is comparable to other assemblers while the rate of false positives - which has been our main focus - is lower. When working in absence of any reference a reasonable trade-off between sensitivity and accuracy is very important for the all the subsequent analyses that have to be performed on results. Indeed false positive reconstructions may lead to biased biological interpretation of results as – for example – they might lead to an overestimation of “novel” transcripts.

In addition, STAble was designed to be parallelizable and grid-friendly, allowing to perform the computationally onerous assembly task even in absence of dedicated infrastructures: is quite surprising that in one of the test scenarios existing assemblers failed with 48GB of RAM while STAble was able to run on a desktop PC.

STAble was successfully integrated with a new analysis workflow based on metabolic model network recently described in [12]. The combination of STAble with this workflow can be used as an “expert system” to obtain more punctual information about the metabolic pathways activated in a bacterial community. The same level of information is not fully available when using only metagenomics and even meta-transcriptomics data.

## Conclusions

Metatranscriptomics is the community based evolution of RNA-Seq analysis and might represent a critical step to further elucidate the role of complex microbial communities in their environment and in the physiology and pathology of host organisms. From a clinical perspective most of the evidence so far accumulated (and that can be collected from standard metagenomics studies) is linked to the role of specific species, genera or families rather than their metabolic output. While this might be optimal in terms of impact on immune recognition, immune education and trigger of autoimmune processes, this approach may be insufficient to fully elucidate the impact of microbial communities on processes such as metabolic diseases, inflammatory response, and nutrient availability which are potentially more strictly related to the global metabolic output rather than to the phylogenesis of the species composing a specific microbiota.

Integrating a robust assembler for metatranscriptomic data and expanding its informative potential with the integration of a metabolic model network could be an improved tool to characterize actively transcribed genes in a microbial community and to predict their metabolic output.

## Abbreviations

FPA: False Positive class A
FPB: False Positive class B
HMY: High Methane Yield
KEGG: Kyoto Encyclopedia of Genes and Genomes LMY Low Methane Yield
NGS: Next Generation Sequencing
RNA-seq: RNA sequencing
SRA: Sequence Read Archive
